# Diagnostic value of Pentraxin-3 in patients with sepsis and septic shock in accordance with latest sepsis-3 definitions

**DOI:** 10.1186/s12879-017-2606-3

**Published:** 2017-08-09

**Authors:** Sonja Hamed, Michael Behnes, Dominic Pauly, Dominic Lepiorz, Max Barre, Tobias Becher, Siegfried Lang, Ibrahim Akin, Martin Borggrefe, Thomas Bertsch, Ursula Hoffmann

**Affiliations:** 1 First Department of Medicine, University Medical Centre Mannheim (UMM), Faculty of Medicine Mannheim, University of Heidelberg, Theodor-Kutzer-Ufer 1–3, Mannheim, 68167 Germany; 2 Institute of Clinical Chemistry, Laboratory Medicine and Transfusion Medicine, General Hospital Nuremberg, Paracelsus Medical University, Nuremberg, Germany

**Keywords:** Sepsis, Septic shock, Pentraxin-3, ICU, Biomarkers, Sepsis-3

## Abstract

**Background:**

Pentraxin-3 (PTX-3) is an acute-phase protein involved in inflammatory and infectious processes. This study assesses its diagnostic and prognostic value in patients with sepsis or septic shock in a medical intensive care unit (ICU).

**Methods:**

The study includes 213 ICU patients with clinical criteria of sepsis and septic shock. 77 donors served as controls. Plasma levels of PTX-3, procalcitonin (PCT) and interleukin-6 were measured on day 1, 3 and 8.

**Results:**

PTX-3 correlated with higher lactate levels as well as with APACHE II and SOFA scores (*p* = 0.0001). PTX-3 levels of patients with sepsis or septic shock were consistently significantly higher than in the control group (*p* ≤ 0.001). Plasma levels were able to discriminate sepsis and septic shock significantly on day 1, 3 and 8 (range of AUC 0.73–0.92, *p* = 0.0001). Uniform cut-off levels were defined at ≥5 ng/ml for at least sepsis, ≥9 ng/ml for septic shock (*p* = 0.0001).

**Conclusion:**

PTX-3 reveals diagnostic value for sepsis and septic shock during the first week of intensive care treatment, comparable to interleukin-6 according to latest Sepsis-3 definitions.

**Trial registration:**

NCT01535534. Registered 14.02.2012

## Background

Sepsis is a widely common clinical syndrome, caused by a systemic infection and accompanied by consecutive organ failure often leading to a lethal outcome [1–3]. Hence, the early diagnosis and identification of sepsis patients is essential, as early evidence-based treatment and therapeutic interventions are likely to improve survival and decrease in-hospital mortality rates [2, 4–8]. Both the widely used C reactive protein (CRP) and [9] procalcitonin (PCT) are inconsistent concerning diagnostic capacity [10]. Accordingly, the evaluation of new diagnostic biomarkers discriminating patients with sepsis or septic shock in an early stage is essential, as mortality rates of specifically septic shock are still unacceptably high despite the developments of modern intensive care medicine.

Pentraxin-3 (PTX-3) is an acute phase protein representing the long pentraxin subfamily [11–13]. Production of PTX-3 is strongly induced by cytokines like interleukin-1, tumor necrosis factor α (TNF-α) and by toll-like receptor (TLR) agonists, but not by interleukin 6 (IL-6) or interferons [11, 12]. PTX-3 is expressed in various cells, like dendritic cells, monocytes, endothelial cells or neutrophils during inflammatory processes [13–15]. Several studies found increased PTX-3 expression due to various specific infectious agents such as aspergillus fumigatus, *Staphylococcus aureus*, *pseudomonas aeruginosa*, *Klebsiella pneumoniae*, *E. coli*, *neisseria meningitides*, and multiple viruses [16–21]. PTX-3 appears to reveal significant potential as a novel early diagnostic and prognostic biomarker in infectious disorders and septic patients as analyzed in former studies [13–16, 22, 23]. However, the previous studies showed inconsistent study cohorts of different size and composition as well as different follow-up periods, not enabling an expressive conclusion considering the role of PTX-3 in these patients [23–31].

Additionally, there are currently no biomarker studies evaluating the diagnostic value of PTX-3 according to the latest Sepsis-3 definitions [3]. Therefore, this study applies these definitions and aims to investigate the diagnostic value of PTX-3 in patients with sepsis and septic shock during the first week of intensive care treatment.

## Methods

### Study patients, design and data collection

The Mannheim Sepsis Study (MaSep, clinicaltrials.gov identifier: NCT01535534) was conducted as a mono-centric prospective controlled study at the University Medical Centre Mannheim (UMM), Germany. Patient enrolment started in October 2011. The study was carried out according to the principles of the declaration of Helsinki and was approved by the medical ethics commission II of the Faculty of Medicine Mannheim, University of Heidelberg, Germany. Written informed consent was obtained from each participating patient or their legal representatives. The study was designed to reflect a representative cohort of patients found at an internal intensive care unit (ICU) with a minimum age of 18 years and proven criteria of sepsis or septic shock [32]. Main exclusion criteria were any traumatic or postoperative cause of sepsis development. We enrolled a total of 30 healthy volunteers in addition to 30 hospitalized patients being treated for different medical conditions without any evidence of infection (normal CRP, WBC, body temperature) serving as controls.

At the end of each hospital treatment, two study physicians independently reviewed all available clinical data of the study patients in order to determine each patient’s correct disease severity on each day. For the present analysis the latest sepsis-3 definitions of 2016 (i.e. sepsis, septic shock) were applied and all patients were re-classified according to these new definitions [3]. A minor number of patients had to be down classified, as in retrospect; they did not fulfill the criteria for sepsis nor septic shock on day 1 (*n* = 17). They were therefore additionally merged with the 60 controls. Naturally, over the course of ICU treatment, patients improved or deteriorated. This means a number of patients, initially presenting with septic shock, could improve to sepsis or even better between days 3 and 8, while patients with sepsis on day 1 could develop a septic shock on day 3 or 8. Therefore, on days 3 and 8 the distribution of patients per group is different than on day 1.

The criteria for sepsis and septic shock were as follows [3]: Patients were assigned to the sepsis-group if an increase of the Sequential Organ Failure Assessment (SOFA) score of 2 points or more was observed as a consequence to a present infection. When patients additionally had a persisting hypotension with vasopressor requirement to maintain a mean arterial pressure of at least 65 mmHg and the serum lactate level was greater than 2 mmol/l despite volume resuscitation, they were classified as septic shock.

In the formerly established criteria, there was an additional definition for *severe sepsis*, which is now met by the new criteria for sepsis, as well as for the *systemic inflammatory response syndrome (SIRS)*, which was diagnosed if at least two of the following symptoms were present: body temperature ≥ 38 °C or ≤36 °C, heart rate ≥ 90 beats per minute, tachypnea (respiratory rate ≥ 20/min or hyperventilation: PaCO2 ≤ 32 mmHg) and leukocytosis (≥12,000/cu mm) or leukopenia (≤4000/cu mm) [33].

According to new guidelines, lactate levels were assessed for all patient groups [3, 34]. Disease severity on the ICU was documented by the acute physiology and chronic health evaluation II (APACHE II) and the sequential organ failure assessment (SOFA) score [35, 36].

All patient data, such as creatinine levels, hemoglobin, hematocrit, white blood cell count, platelet count, CRP, bilirubin, sodium, potassium, urea, interleukin 6 (IL-6), PCT, body temperature, respiratory rate, heart rate, blood pressure, partial pressure of O2 and CO2, bicarbonate, base excess, lactate, pH value, Glasgow coma scale (GCS) were documented from the patient files. Additionally, prior medical history, age, sex, body weight and the germ spectrum were documented.

Blood samples for PTX-3 measurements were taken within 24 h after clinical onset of sepsis or septic shock on the ICU (day 1) as well as on day 3 and 8 of ICU treatment. All patients were followed up until 30 days and 6 months after study inclusion by direct telephone visits with the patients or their general practitioners. The main prognostic outcome was all-cause mortality after 30 days and 6 months.

### Biomarker measurements

Blood samples were obtained by venipuncture into serum and ethylenediaminetetraacetic acid (EDTA) monovettes® (SARSTEDT AG & Co.; Nümbrecht, Germany). Within 30 min all blood samples were centrifuged at 2500×g at 20 °C for 10 min. Plasma and serum were separated and aliquoted. The aliquoted samples were cooled down with liquid nitrogen before being stored at -80 °C until Analysis.

PTX-3 measurements were performed with the Quantikine® Human Pentraxin 3/TSG-14 Immunoassay (R&D Systems Inc., Minneapolis, USA) using plasma from EDTA monovettes®. IL-6 and PCT were measured in serum. IL-6 was measured with reagents from Roche Diagnostics (Roche Diagnostics, Mannheim, Germany) and PCT was measured with reagents from Thermo Fisher Scientific (Thermo Fisher Scientific Clinical Diagnostics, BRAHMS GmbH, Henningsdorf, Germany). The assays were performed on a Cobas e601 twin module and on a Cobas e602 module (Roche Diagnostics, Mannheim, Germany). Interleukin-6 and PCT measurements were performed at the central laboratory in Nuremberg, Germany.

### Statistical analysis

Statistical analysis was performed with InStat (GraphPad Software) and SPSS software (SPSS Software GmbH). Comparisons between two groups, for instance healthy subjects and patients with sepsis, were performed with the Student’s t-test. In case of more than two groups, metric variables were compared by analysis of variance (ANOVA), if applicable. For normally distributed metric data (as tested by the Kolmogorov-Smirnov test), the Student t-test was applied. For variables not normally distributed, the Mann–Whitney U-test was used as a nonparametric test. Spearman’s rank correlation for nonparametric data was used to test the association of PTX-3 blood levels with medical parameters. Qualitative parameters were analyzed by use of a 2 × 2 contingency table and Chi2 test or Fisher’s exact test as appropriate. Quantitative data are presented as mean ± standard error of mean (SEM) or as median and interquartile ranges (IQR) (i.e. 25th to 75th percentiles), depending on the distribution of the data. For qualitative parameters absolute and relative frequencies are presented. A test for linear trend was applied to compare the biomarker levels in the different groups of disease severity. All analyses were exploratory and a two-tailed *p*-value of < 0.05 was taken as a cut-off for statistical significance.

### Diagnostic value of biomarkers

For C-statistics: To evaluate pre-test discriminative capacities of each biomarker, receiver-operating characteristic (ROC) curve analyses were performed with calculation of area under the curve (AUC) for diagnosis of sepsis and septic shock during the first week of ICU treatment at days 1, 3 and 8 for each biomarker. For PTX-3, diagnostic goodness criteria (that is, accuracy, specificity, sensitivity, negative/positive predictive values (NPV/PPV), and relative risk) were calculated. Accuracy was defined as the sum of true positives plus true negatives divided by all measured patients.

## Results

Baseline characteristics are presented in Table 1. A total of 213 patients and 77 healthy controls have been enrolled into the MaSep study. At the time of enrollment, 34% of patients suffered from sepsis and 66% from septic shock, respectively. The most common site of infection were the lungs (approximately 62%) and abdomen (12%).

**Table 1:** Baseline characteristics of the Mannheim Sepsis Study (MaSep) at day 1

	Controls (*n* = 77)	Sepsis (*n* = 73)	Septic Shock (*n* = 140)	*p-values*
Age, years (mean, range)	64 (42–87)	65 (26–88)	67 (39–87)	*0.9*
Gender, n (%)				
Male	42 (55)	47 (64)	101 (72)	*<0.001*
Female	35 (45)	26 (36)	39 (28)	*0.1*
Site of infection, n (%)				
Lung	-	47 (64)	84 (60)	*0.001*
Urinary tract	-	9 (12)	12 (8)	*0.5*
Abdominal	-	10 (14)	15 (11)	*0.3*
Central nervous system	-	0 (0)	0 (0)	*-*
Skin	-	2 (3)	8 (6)	*0.06*
Heart	-	4 (6)	4 (3)	*1.0*
Blood	-	1 (1)	10 (7)	*0.007*
Others	-	0 (0)	7 (5)	*-*
Laboratory values, mean ± SEM				
White blood cells (10^9^/L)	-	16.1 ± 1.4	18.1 ± 1.2	*0.4*
Platelets, (10E9/L)	-	221 ± 16.2	169 ± 9.9	*0.01*
Bilirubin, mg/dl	-	1.6 ± 0.4	3 ± 0.6	*0.004*
Lactate mg/dl	-	2.3 ± 0.2	3.5 ± 0.3	*0.002*
Creatinine, mg/dl	-	2.4 ± 0.2	2.8 ± 0.2	*0.05*
C reactive proteine, mg/l	-	186 ± 12.5	207 ± 9.5	*0.2*
Procalcitonin, ng/ml	-	12.5 ± 2.7	31 ± 5.7	*0.002*
Interleukin 6, pg/ml	-	16,281 ± 15,560	6802 ± 1911	*<0.001*
pCO2 (mmHg)	-	37.9 ± 1.6	43.7 ± 1.6	*0.02*
Positive blood cultures, n (%)	-	28 (38)	62 (44)	*<0.001*
ICU parameters, mean ± SEM				
ICU days	-	10 ± 1.1	14.6 ± 1.3	*0.1*
Ventilation days	-	4.3 ± 0.9	9.4 ± 1.1	*<0.001*
Catecholamine days	-	2 ± 0.5	7.4 ± 0.6	*<0.001*
Renal replacement therapy days	-	1 ± 0.3	4.2 ± 0.7	*<0.001*
APACHE II, mean ± SEM	-	20.5 ± 0.9	27.6 ± 0.6	<0.001
SOFA score, mean ± SEM	-	7.2 ± 0.4	12.3 ± 0.3	<0.001

### Distribution of PTX-3 according to latest sepsis-3 definitions

**Fig. 1:**
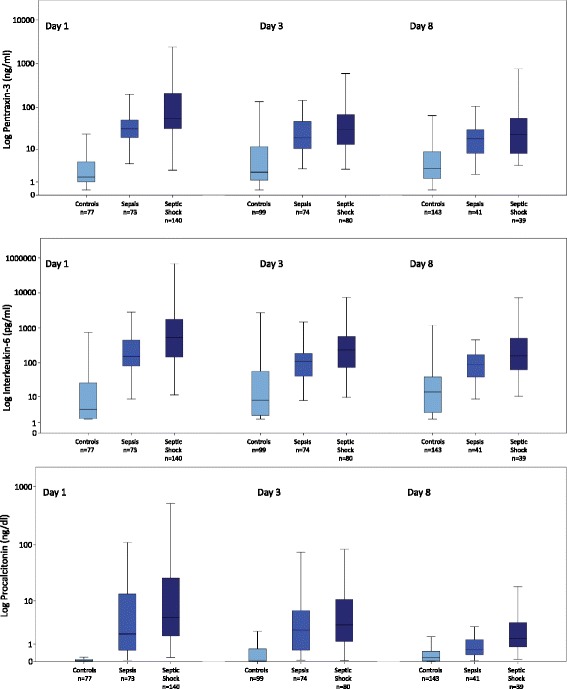
PTX-3 (*top*), procalcitonin (PCT, *middle*) and IL-6 plasma levels (*bottom*) in patients admitted to an internal ICU with proven criteria of sepsis and septic shock. Left diagrams show results of biomarker measurements at day 1, middle diagrams show results at day 3 and right diagrams show results at day 8. Seventy-seven individuals served as a control group at day 1. Data are presented as medians with 25th and 75th percentiles (boxes) and 5th and 95th percentiles (whiskers)

Figure 1 shows the distribution of PTX-3, IL-6 and PCT in the different groups of disease severity on day 1, 3 and 8. On each day of measurement, PTX-3 levels of patients with sepsis as well as levels of patients with septic shock were significantly higher than in the control group (*p* ≤ 0.001). Although overall PTX-3 levels showed a decreasing linear trend from day 1 to day 8 of ICU treatment (*p* = 0.0001), median serum PTX-3 levels in patients with septic shock consistently remained higher than in patients with sepsis throughout the first week of ICU treatment.

**Fig. 2:**
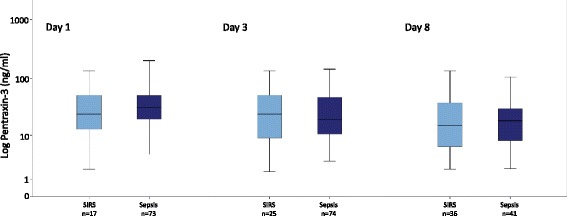
PTX-3 levels in patients admitted to an internal ICU with proven criteria of SIRS and sepsis on day 1 (*left*), 3 (*middle*) and 8 (*right*). Data are presented as medians with 25th and 75th percentiles (boxes) and 5th and 95th percentiles (whiskers)

Within the control cohort, 17 individuals fulfilled the former criteria for SIRS on day 1 (n_d3_ = 25, n_d8_ = 36). Figure 2 demonstrates the PTX-3 levels in this cohort compared to patients with sepsis. PTX-3 levels on day one were higher in the sepsis cohort (median 31.4 ng/ml) compared to the SIRS cohort (median 23.8 ng/ml), however not statistically significant (*p* > 0.05). Accordingly, no significant difference was shown on days 3 and 8 between both subgroups.

### Univariate correlations of PTX-3 levels

Statistically significant correlations of PTX-3-levels were shown with the following clinical parameters (Table 2): higher lactate levels (*r* = 0.36, *p* < 0.0001), APACHE II score (*r* = 0.36, *p* < 0.0001), a higher SOFA score (*r* = 0.36, *p* < 0.0001) as well as with a lower mean arterial pressure (*r* = −0.25, *p* < 0.0001) and higher serum creatinine (*r* = 0.17, *p* < 0.01). There was also a significant association of PTX-3 with the established inflammatory biomarkers IL-6 (*r* = 0.37, *p* < 0.0001), PCT (*r* = 0.28, *p* < 0.0001), and CRP (*r* = 0.26, *p* < 0.0001).

**Table 2:** Univariate correlations of PTX-3 with laboratory and clinical parameters in all patients (n=213) at day 1

	r	*p* value
Creatinine	0.17	0.013
Bilirubin	0.22	0.003
Lactate	0.36	<0.001
Platelets	−0.19	0.005
C reactive proteine (CRP)	0.26	<0.001
Procalcitonin (PCT)	0.28	<0.001
Interleukin 6	0.37	<0.001
Mean arterial pressure	−0.25	<0.001
Mechanical ventilation days	0.17	0.011
Catecholamines days	0.18	0.009
SAPS II score	0.27	<0.001
APACHE II score	0.36	<0.001
SOFA score	0.36	<0.001

### PTX-3 discriminates sepsis and septic shock according to latest sepsis-3 definitions

**Fig. 3:**
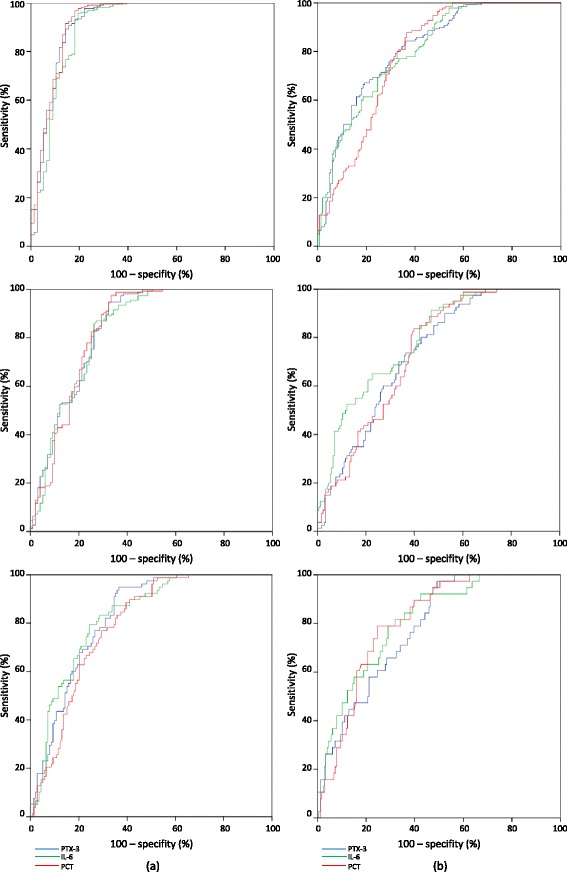
Receiver-operating characteristic (ROC) curves revealing valuable discrimination of patients with sepsis (**a**) and septic shock (**b**) by serum levels of PTX-3, IL-6 and PCT on day 1, 3 and 8 (from top to *bottom*)

C-statistics revealed valuable diagnostic capacity for PTX-3 (Table 3). Diagnostic AUCs for discriminating patients with at least sepsis were statistically significant on each day of measurement (*p* = 0.0001) (Fig. 3a). PTX-3 levels valuably discriminated the presence of at least sepsis on each day (minimal AUC = 0.82) (Fig. 3a) as well as of septic shock (minimal AUC = 0.73) (Fig. 3b) and was overall comparable to IL-6 and PCT (Table 3).

**Table 3:** Discriminative capacitites of biomarkers for diagnosis of sepsis severity at days 1, 3 and 8 of ICU treatment, analyzed as area under the curves (AUCs (95% CI))

	Pentraxin-3	Interleukin-6	Procalcitonin	CRP	White blood cells
Day 1					
≥ Sepsis (*n* = 213)	0.92 (0.87–0.97) *p = 0.0001*	0.91 (0.86–0.95) *p = 0.0001*	0.92 (0.88–0.97) *p = 0.0001*	0.82 (0.72–0.91) *p = 0.0001*	0.59 (0.46–0.72) *p* = 0.2
Septic shock (*n* = 140)	0.81 (0.77–0.86) *p = 0.0001*	0.81 (0.76–0.85) *p = 0.0001*	0.79 (0.74–0.84) *p = 0.0001*	0.61 (0.53–0.68) *p = 0.008*	0.55 (0.47–0.62) *p* = 0.2
Day 1: controls *n* = 77; sepsis *n* = 73; septic shock *n* = 140.					
Day 3					
≥ Sepsis (*n* = 154)	0.84 (0.79–0.90) *p = 0.0001*	0.84 (0.78–0.89) *p = 0.0001*	0.84 (0.78–0.90) *p = 0.0001*	0.70 (0.60–0.79) *p = 0.0001*	0.53 (0.42–0.63) *p* = 0.6
Septic shock (*n* = 80)	0.73 (0.67–0.80) *p = 0.0001*	0.79 (0.73–0.85) *p = 0.0001*	0.74 (0.68–0.80) *p = 0.0001*	0.69 (0.61–0.76) *p = 0.001*	0.54 (0.46–0.63) *p* = 0.3
Day 3: controls *n* = 99; sepsis *n* = 74; septic shock *n* = 80.					
Day 8					
≥ Sepsis (*n* = 80)	0.82 (0.77–0.88) *p = 0.0001*	0.83 (0.77–0.88) *p = 0.0001*	0.79 (0.74–0.85) *p = 0.0001*	0.74 (0.66–0.82) *p = 0.0001*	0.66 (0.57–0.75) *p = 0.001*
Septic shock (*n* = 39)	0.79 (0.72–0.85) *p = 0.0001*	0.81 (0.75–0.88) *p = 0.0001*	0.81 (0.75–0.87) *p = 0.0001*	0.70 (0.60–0.79) *p = 0.0001*	0.74 (0.65–0.83) *p = 0.0001*
Day 8: controls *n* = 143; sepsis *n* = 41; septic shock *n* = 39.					

A minimal AUC was set at ≥0.75 (highlighted in bold type). Significant *p* values (<0.05) are in italic type

In addition, CRP also showed statistically significant AUCs to discriminate sepsis and septic shock on each day. However the AUCs of CRP were consistently lower than those of PTX-3 throughout the first week of ICU treatment. The white blood cell count was not able to discriminate sepsis or septic shock on day 1 or 3 and revealed only moderate diagnostic value on day 8 with AUCs below 0.75 (Table 3).

Subsequently, uniform diagnostic cut-offs were defined to diagnose at least sepsis (≥5.0 ng/ml) and septic shock (≥9.0 ng/ml) (Table 4), while trying to maintain a sensitivity of at least 70% on each day of measurement, with focus on the first day to enable an early diagnosis. Specifically for the presence of sepsis, the minimal sensitivity was 92% with an according negative predictive value of at least 89%, throughout all treatment days 1, 3 and 8.

**Table 4:** Diagnostic goodness criteria of Pentraxin-3 for diagnosis of sepsis and septic shock during the first week of ICU treatment

	AUC	cutoff (ng/ml)	accuracy (%)	sensitivity (%)	specificity (%)	PPV (%)	NPV (%)	relative risk	*p* value
Day 1									
≥ Sepsis	0.92	5	92	98	79	92	93	13.6	0.0001
≥ Septic shock	0.81	9	68	93	45	61	87	4.8	0.0001
Day 3									
≥ Sepsis	0.84	5	84	95	67	82	89	7.6	0.0001
≥ Septic shock	0.73	9	61	85	50	44	88	3.6	0.0001
Day 8									
≥ Sepsis	0.82	5	74	92	64	59	94	9.4	0.0001
≥ Septic shock	0.79	9	66	72	65	31	92	3.6	0.0001

*AUC Area under curve, PPV and NPV positive and negative predictive values*

## Discussion

The present study evaluates the diagnostic value of PTX-3 in patients with sepsis and septic shock according to the latest Sepsis-3 definitions during the first week of intensive care treatment. Since sepsis and septic shock are usually diagnosed inadequately and delayed, current guidelines still demand time-dependent risk stratification of each individual patient during intensive care treatment. Accordingly, diagnostic assessment of study patients was performed exemplarily on day 1, 3 and 8 of ICU treatment.

The study revealed that valuable and consistent discrimination of sepsis and septic shock was achieved by measurements of PTX-3 plasma levels during days 1, 3, and 8 of ICU treatment, specifically for the presence of at least sepsis. In addition, PTX-3 correlated with disease severity and degree of organ dysfunctions as assessed by clinical scores such as the SOFA score, as has also been described in earlier studies [24, 31]. In line with previous studies, this study confirms that circulating PTX3 concentrations are elevated in sepsis and even higher in septic shock [31]. Consistent with the current observations, multiple previous studies presented significant and valuable AUCs for discriminating sepsis or septic shock from healthy controls [24, 28, 29]. The diagnostic superiority of PTX-3 over PCT or CRP is still under debate, as different studies so far have been inconsistent regarding their diagnostic capacity in patients with sepsis and septic shock, applying different criteria and definitions of the sepsis syndrome [24, 28]. No study is currently available applying the newest Sepsis-3 definitions for novel biomarker analyses.

In contrast to previous studies, the present one aimed to evaluate the potential diagnostic value of PTX-3 in a real-life setting during the first week of treatment on a medical ICU, representing the most critical time frame of patients suffering from sepsis or septic shock [5, 6, 37]. Combining PTX-3 with established biomarkers, such as IL-6, might be of additional value for the discrimination of sepsis and septic shock. Notably, the presented detailed correlations and comparisons of PTX-3 with other established clinical parameters have only scarcely been described yet [31]. The ongoing systemic activation of inflammatory biomarkers such as CRP, interleukin-6 and PCT during sepsis and septic shock may be an explanation of their correlation with PTX-3 levels in affected patients [38, 39]. PTX-3 itself is known to be induced by several cytokines leading to a rapid synthesis in-vitro [11, 12]. Specifically the stimulation by interleukin-1 and TNF-α was shown to increase plasma levels of PTX-3. This effect was shown in various cells and inflammatory steps, such as during the initial phase of inflammation by recognizing pathogens and activation of the complement pathway, thus allowing an approach to explain the early elevated PTX-3 plasma levels in patients with infection [11, 12, 23].

PTX-3 levels revealed no significant difference between patients with SIRS and sepsis in the present cohort. Previous studies measured PTX-3 levels in both groups, however did not compare the values for statistical significance [24]. Additionally it should be noted, that SIRS is considered inadequately specific and sensitive and is no longer part of the sepsis definitions [3].

The strengths of the present study were its prospective character, augmenting the current knowledge about PTX-3 by evaluating its diagnostic capacity at different time points of the first week of ICU treatment. Additionally, this study provides the first analysis of PTX-3 within the individual subgroups sepsis and septic shock as classified by implementing the recently updated sepsis-3 definitions [3]. It is important to identify these high risk patients as they are endangered of fatal outcome even within the following months after hospital discharge [38]. Novel biomarkers such as PTX-3 in combination with established ones such as IL-6 might improve the individual time-dependent risk-stratification of patients with sepsis or septic shock.

### Limitations

The present study was performed as a single centre study. Analyses were not blinded to white blood cells and CRP, as both are used in daily routine by the practicing clinicians. After retrospective re-evaluation of main diagnoses, a minor part of the patients was downgraded to controls. Only patients with an infection and an increase in SOFA Score were included in the study, not allowing a statement on the differentiation between an infection without increase in SOFA score and patients with sepsis. Upcoming randomised controlled multi-centre studies might verify the results of the present study.

## Conclusions

In summary, PTX-3 valuably discriminates the different stages of sepsis severity during the first week of intensive care treatment according to latest Sepsis-3 definitions.

## Key points

- PTX-3 was evaluated in 213 medical ICU patients suspected to suffer from at least sepsis.
- PTX-3 discriminates valuably sepsis and septic shock from healthy controls corresponding to uniform cutoff levels.
- PTX-3 was evaluated according to the latest Sepsis-3 definitions.

## Abbreviations

APACHE: Acute Physiology And Chronic Health Evaluation
AUC: Area under the curve
CRP: C - reactive protein
ICU: Intensive care unit
IL-6: Interleukin-6
IQR: Interquartile range
PCT: Procalcitonin
PTX-3: Pentraxin-3
ROC: Receiver-operating characteristic
SIRS: Systemic inflammatory response syndrome
SOFA: Sepsis-related Organ Failure Assessment
WBC: White blood cells
